# Brain–computer interface to predict impulse buying behavior using functional near-infrared spectroscopy

**DOI:** 10.1038/s41598-022-22653-8

**Published:** 2022-10-26

**Authors:** SuJin Bak, Yunjoo Jeong, Minsun Yeu, Jichai Jeong

**Affiliations:** 1 Department of Brain and Cognitive Engineering, Korea University, Seoul, 02841 South Korea; 2 Center for Research in Marketing in School of Business at Korea University, Seoul, 02841 South Korea; 3 College of Business Administration, University of Ulsan, Ulsan, 44610 South Korea

**Keywords:** Predictive markers, Cognitive control

## Abstract

As the rate of vaccination against COVID-19 is increasing, demand for overseas travel is also increasing. Despite people’s preference for duty-free shopping, previous studies reported that duty-free shopping increases impulse buying behavior. There are also self-reported tools to measure their impulse buying behavior, but it has the disadvantage of relying on the human memory and perception. Therefore, we propose a Brain–Computer Interface (BCI)-based brain signal processing methodology to supplement these limitations and to reduce ambiguity and conjecture of data. To achieve this goal, we focused on the brain’s prefrontal cortex (PFC) activity, which supervises human decision-making and is closely related to impulse buying behavior. The PFC activation is observed by recording signals using a functional near-infrared spectroscopy (fNIRS) while inducing impulse buying behavior in virtual computing environments. We found that impulse buying behaviors were not only higher in online duty-free shops than in online regular stores, but the fNIRS signals were also different on the two sites. We also achieved an average accuracy of 93.78% in detecting impulse buying patterns using a support vector machine. These results were identical to the people's self-reported responses. This study provides evidence as a potential biomarker for detecting impulse buying behavior with fNIRS.

## Introduction

International tourism has declined as mobility is limited because of the COVID-19 pandemic. However, international travel restrictions are gradually easing as more people chose to get vaccinated against COVID-19. In line with this trend, the paradigm of the tourism industry has changed, and various tourism activities are resuming to alleviate the damage caused by COVID-19. Travelers regard shopping as an essential tourism activity. The first or last place for travelers to shop is from a duty-free shop. Since only inbound and outbound travelers can use duty-free shops, they can be differentiated from general shopping malls where anyone can shop. Travelers’ unplanned expenditures occur when the environment in duty-free shops, such as luxurious interiors, famous brands, and new products meet their expectations and excitement^1^. We define these unplanned expenditures as impulse buying behavior^2^.

Due to the special and unusual environment in duty-free shops, people tend to engage in impulse buying^3^. Thus, impulse buying is an important phenomenon in the situation of duty-free shopping. To understand the impact of this impulse buying phenomenon by people, several studies have identified important determinants such as the joy of the trip itself, time pressure, and price discount^4–6^. First, impulse buying is associated with the joy of the travel itself such as the expected positive effect^5^. Given that the positive effects such as enthusiasm, activeness, and pleasant engagement have a positive impact on impulsive buying^5^, we predict that the joy of the travel will encourage impulse buying behavior. Second, time pressure affects people’ decision-making, which can control the effect of impulse buying behavior according to the level of time pressure^7^. Therefore, time pressure affects the psychology of those who have to finish shopping at duty-free shops within a limited time, which can encourage impulse buying behavior. Third, people prefer shopping at duty-free shops that sell at discounts than the original price^8^. When prices are lowered, people spend less than the original price, and the perceived price discount pleases the travelers, which is a contributing factor to their impulse buying behavior. Hence, people have a high preference for shopping at duty-free shops that sell products at discounted prices, unlike general shopping malls where anyone can shop. However, these three determinants are influenced by the unconscious behavior of people. Past studies have demonstrated that impulse buying is an unconscious behavior, which arises from a sense of out-of-control^9^. We believe that BCI technique has become a key to understanding people' impulse buying behavior based on their unconsciousness.

BCI appears to be useful for closely monitoring people’s unconscious behavior^10^. It is defined as a direct interaction between the brain and external devices, which involves exploring human cognitive or sensory-motor functions and identifying functions for each part of the brain^11^. Especially, many researchers have used BCI-based techniques to identify the brain mechanisms underlying people’s behavior and decision-making^12^. Many researchers recognize the importance of BCI in light of these trends but there is a still lack of empirical evidence. Furthermore, traditional studies have limitations in focusing only on the factors (e.g., trip itself, time pressure, and price discounts) influencing impulse buying behavior^13^. Existing studies have examined impulse buying behavior using data obtained from self-reported measurements, which mostly depend on the people’s memories of their own experiences. These studies must subjectively rely on the people's feelings and think, which are not absolute^14^.

These problems can be solved by identifying brain functions. Especially, impulsiveness can be interpreted according to the brain’s prefrontal cortex (PFC) activity^15^. PFC is a part of the brain located at the front of the frontal lobe. The most important PFC function is the executive function, which includes decision-making, planning, and cognitive control of one’s attention, behavior, and thoughts^16^. Thus, analyzing the brain activity of PFC makes the mechanism of impulse buying behavior predictable. The PFC has four sub-regions; the dorsolateral prefrontal cortex (DLPFC), ventrolateral prefrontal cortex (VLPFC), medial prefrontal cortex (mPFC), and orbitofrontal cortex (OFC). The four activated regions of interest (ROIs) have different functions. Several studies have reported that the DLPFC, VLPFC, and OFC are found to exhibit activity related to impulse suppression^17^. In contrast, some studies have presented experimental evidence for the involvement of the medial prefrontal cortex (mPFC) in reward-related mechanisms in the brains of humans^18,19^. Recently, several studies have found that the left VLPFC is partially involved in the reward systems^20^. The reward systems are closely related to impulsiveness. Impulsive people have structural abnormalities in brain regions associated with reward sensitivity processing in accordance with^21^. Hence, ROIs’ analysis helps to understand impulse buying behavior.

To identify the brain function and analyze the brain activation for the subregions within the PFC, the BCI technology can be utilized. There are representative non-invasive neuroimaging devices such as electroencephalogram (EEG)^22^, functional magnetic resonance imaging (fMRI)^23^, and functional near-infrared spectroscopy (fNIRS)^24^ in which brain activation patterns are measurable^25^. Among them, we introduced fNIRS, the latest optical near-infrared biosensor for measuring brain activity. Table 1 gives an overview of recent research using fNIRS. fNIRS is at the intersection of each disadvantage of fMRI and EEG. Their measurement performance may be influenced by temporal resolution (i.e., a scale of time required to obtain data from the same location) or spatial resolution (i.e., a scale of a spatial domain that can be expressed in detail). The smaller the temporal and spatial resolution, the greater the amount of obtainable information and the more detailed it is expressed. fNIRS has better temporal resolution than fMRI but has a limited spatial resolution. Whereas fMRI has better spatial resolution than most commonly used EEG systems, but more limited temporal resolution. fNIRS, which has both the advantages of EEG and fMRI, is excellent for measuring concentration changes in oxygenated hemoglobin (∆HbO) and in deoxygenated hemoglobin (∆HbR) during mental tasks. It has been widely used in cognitive experiments related to impulsiveness, and a recent study has revealed a correlation between impulsiveness and emotional characteristics related to PFC dysfunction^26^. In addition, fNIRS is easier to use and more cost-effective than fMRI.

**Table 1:** Comparison of neuromarketing research on consumer purchasing behavior published over the past four years using fNIRS.

Articles	Issued years	Research specifications for neuromarketing using fNIRS	
		Subject (N)	Experimental paradigms
Krampe, Caspar et al.	2018	36	Buying scenario
Krampe, Caspar et al.	2018	42	Buying scenario for brand
Cakir et al.	2018	33	Buying behavior
Agrali et al.	2018	14	Buying by advertisements
Amanda Sargent et al.	2019	27	Choose a rate plan
Meyerding et al.	2020	31	Buying behavior
A Nissen et al.	2020	24	Neural processing of ecommerce websites
Dual et al.	2021	40	Buying behavior
A Nissen et al.	2021	28	Buying behavior for digital signage
A Nissen et al.	2021	20	Buying behavior
Kim et al.	2021	20	Buying behavior

Therefore, we propose the neuroscientific methodology for BCI technology to predict impulse buying behavior using fNIRS, which is the latest neuroimaging modality. To accomplish this goal, we compared the hemodynamic responses of fNIRS observed in Session 1, which elicited impulse buying behaviors, and Session 2, which is a control task. We also analyzed the brain activation in the PFC (i.e., mPFC), which is closely related to excitement or reward inspired by travel. As a result, we found that impulse buying behavior was not only higher in Session 1 than in Session 2, but the fNIRS signals were also different in the two sessions, statistically. These empirical findings are the same as the results of the participants' self-reported responses. Finally, we detect impulse buying behaviors from non-impulse buying behavior using a support vector machine (SVM), a supervised machine learning algorithm. This study provides promising results for BCI technology by measuring brain activity with fNIRS to enhance understanding of impulse buying patterns.

## Methods

### Participants

We calculated the desired sample size using G*Power 3.1.9.2. before conducting the study. The desired sample size was 30 people when we assumed a moderate effect size (α = 0.05, power = 0.8, effect size = 0.25), according to Ref.^27^. These variables are standardized. Therefore, we selected 30 participants in the experiment (13 males and 17 females, mean and standard deviation of age: M = 25.67 s.d. = 2.69 years). To ensure consistency in brain activation patterns, all the subjects were right-handed and used their dominant hand for performing the experiment. They had normal or corrected-to-normal vision. The participants had no previous history of any physical, mental, or psychological disorders. All participants gave written informed consent to participate in the study, and then they followed COVID-19 safety rules such as wearing masks, social distancing, and using hand sanitizers in accordance with^28^. The experiment was approved by the Korea University Institutional Review Board (KUIRB-2022-0127-01).

### Accquisition of fNIRS signals

**Figure. 1:**
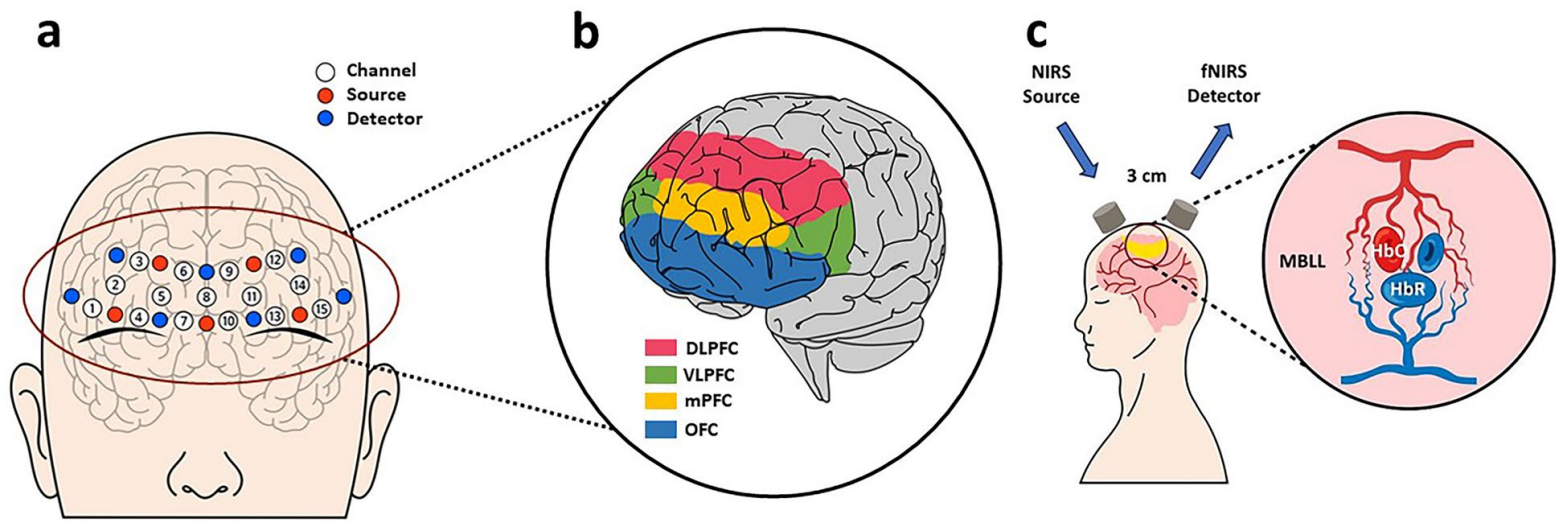
Schematic channel location and measurement principle of fNIRS device: (**a**) indicates the location of fNIRS channels by attaching them to the participants’ forehead. Based on this channel location, the regions of interest (ROI) related to impulsiveness were set as shown in (**b**). (**c**) Shows the measurement principle by fNIRS. We can obtain optical density signals within banana-shaped pathways from detectors. The measured optical density signals are converted into the concentration changes of oxygenated (∆HbO) and deoxygenated (∆HbR) hemoglobin through the modified Beer-Lambert law.

A fNIRS device (NIRSIT Lite, OBELAB Inc., Korea) was used to measure the brain signals at the PFC located in the forehead, which has been fully reliable through many studies^24,29–32^. Figure 1 depicts the fNIRS channel schemes along with concepts of measuring fNIRS between the source and detector. Specifically, Fig. 1a presents the fNIRS channel scheme, which is 15 channels (black open circles), 5 sources (red circles), and 7 detectors (blue circles) in accordance with the 10/20 international system. Centering on channel 8, the right frontal lobe indicates Chs. 1 to 7 and the left frontal lobe indicates Chs. 9 to 15. Figure 1b illustrates the ROIs within the PFC regions related to impulsiveness. The four ROIs consist of DLPFC at the top, VLPFC at both sides, mPFC at the middle, and OFC at the bottom. Through these four ROIs, we investigated the effect of impulse buying behavior on the PFC. Figure 1c presents the concepts of generating fNIRS signals between the source and detector. Each source absorbed near-infrared light of wavelengths 780 nm and 850 nm. Signals were collected from detectors at a sampling rate of 8.138 Hz. The distance between the source and detectors was 3 cm. We can measure optical density signals based on the concept of measuring NIR lights^33^. The raw optical density signals were converted into concentration changes of oxygenated (∆HbO) and deoxygenated (∆HbR) hemoglobin (μM) using the modified Beer–Lambert law^34^. To eliminate physiological artifacts, we carried out digital bandpass filtering in the range of 0.01–0.1 Hz using a 4th-order Chebyshev-II FIR filter that has already demonstrated high signal-to-noise ratio (SNR)^35^. The filtered signals were divided into epochs from 0 to 60 s. Epochs were subjected to a baseline correction, which consisted of subtracting the average value within the reference interval (− 1 to 0 s). The temporal means of ΔHbO in each channel were calculated by averaging the fNIRS data from the task start (0 s) to the task end (60 s) of each epoch. Eventually, we dealt only with ΔHbO signals in subsequent analyses because ΔHbO signals better reflected the hemodynamic activation unlike ΔHbR^36^. The acquired fNIRS dataset and information can be downloaded from https://github.com/SujinBak/Impursiveness.

### Feature extraction and classification for BCI methodology

BCI involves four processing steps such as brain signal acquisition, preprocessing, feature extraction, and classification. Especially, the feature extraction stages have a significant impact on the classification performances such as accuracy. Thus, we adopted the framework according to Park et al.^37^ to investigate the possibility of detecting impulse buying behavior and non-impulse buying behavior. This framework extracted five features: signal mean, signal variance, signal slope, signal kurtosis, and signal skewness. Thus, we obtained the overall data size of 74,400 (i.e., 496 s × 5 features × 30 subjects). All features were rescaled between 0 and 1 to normalize the data size of features.

We used SVM, a supervised learning algorithm, to classify impulse buying and non-impulse buying patterns based on the extracted features. The SVM applied a linear kernel, and sequential minimal optimization (SMO), which automatically optimizes the weights of all the training support vectors. To avoid overfitting, the SVM model was tested using tenfold cross-validation. We divided them into 9 train sets and 1 test set. The SVM model was tested 30 times, and the average accuracy and standard error of the mean (s.e.m) were obtained to measure the outputs’ variability.

### Experimental procedure

**Figure 2:**
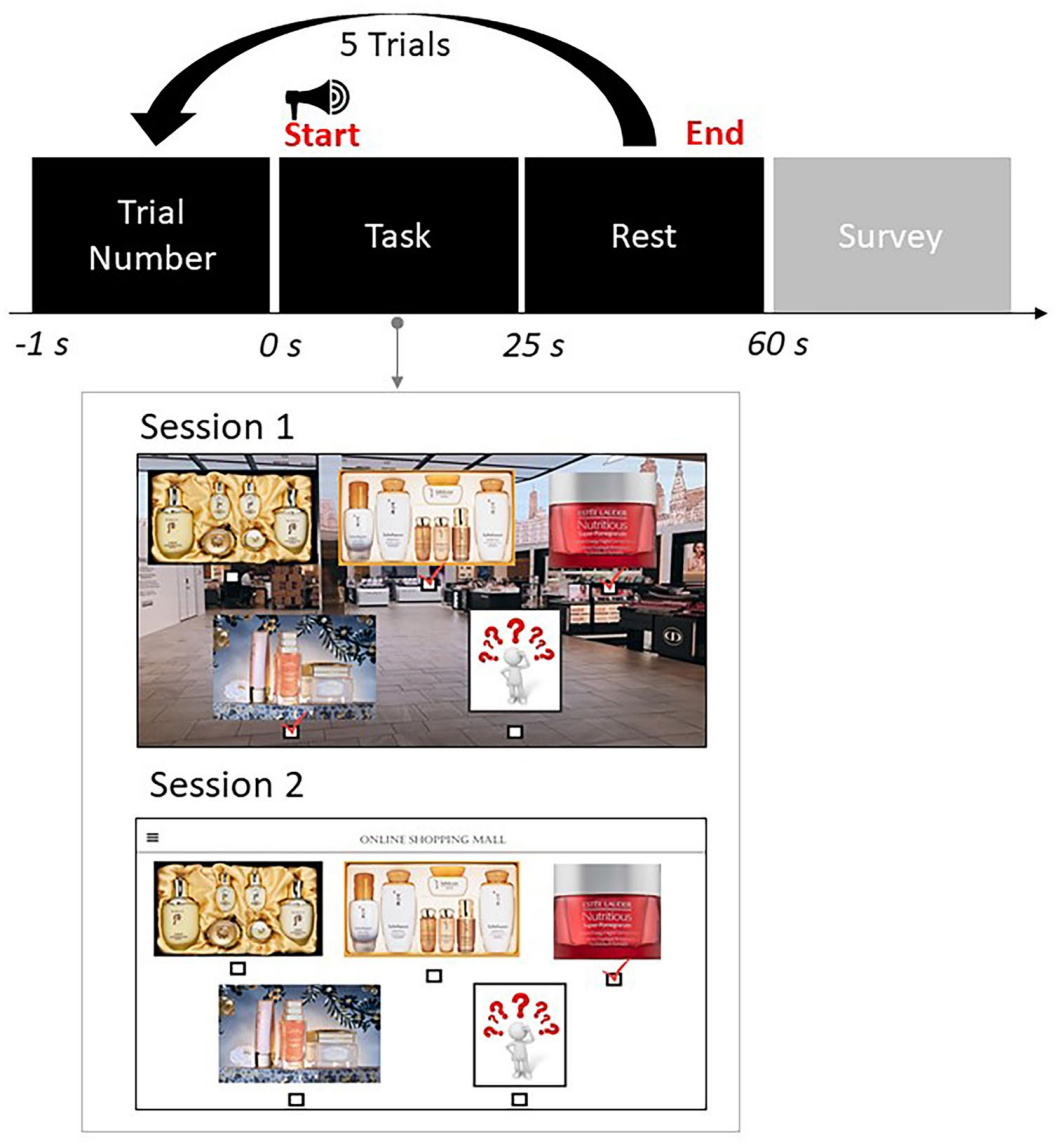
Schematic illustration of the experiment protocol. We instructed the participants to perform Session 1 (the task of shopping at online duty-free shops) and Session 2 (the task of shopping at online shops). After the experiment, a self-reported measurement was conducted.

Two experimental sessions were designed to show brain activation patterns that elicit impulse and non-impulse buying behaviors. To avoid repetitive priming effects, this experiment changes the type of shopping products over 5 repetitions of trials. The selected products consisted of the top five popular categories at online duty-free shops^38^. The product line used in this experiment consists of five categories: cosmetics, alcohol, health supplements, cigarettes, and perfumes, and includes five products in each category. For example, the alcohol category had options such as Glenfiddich, Ballantine’s, Royal Salute, Johnnie Walker, and others. Participants can freely purchase up to five products they want in each category. Each session lasted for 5 min; trial number display (1 s), task (25 s) with a short beep (58.4 dBA, sound level meter, YATO, China), and rest (35 s) periods. Figure 2 presents an overview of the experiment protocol. All subjects can buy enough items that appear in this experiment, but we did not specifically set allowances and earnings because each subject has a different economic situation. We instructed participants to read the following simulated scenarios:

In (a) Session 1, you earn enough money at work or are given an allowance from your parents every month. After you decided to travel abroad, you also get a chance to shop at online duty-free shops, which are more than 30% cheaper than online shopping malls. It's a spontaneous trip, so you can only buy products at online duty-free shops for 20 min. In (b) Session 2, you earn enough money at work or are given an allowance from your parents every month. You have an online shopping mall that you frequently access in your daily life. In both (a) and (b), participants should select whether they would like to purchase the products that appear on the screen.

We used Session 2 as a kind of control group composed of people having normal shopping habits to scrutinize Session 1. Experiments were continuously carried out in the same day in order to maintain the same probe location in each session^24^. The order of the sessions was randomized for each participant and counterbalanced across our study. Lastly, respondents answered their buying impulsiveness propensity and demographics.

### Self-reported measurements

In this study, multiple item scales were used to measure impulse buying behavior. The items in the self-reported measurement were designed based on a review of the following literature^39^: “While shopping for groceries, I have bought more products than what I intended to buy”, “Stock up groceries and/or other necessities”, and “Unusual purchase of groceries”. All respondents answered these items. For the manipulation check of time pressure and price discount, six and three items were measured using a modified multi-item scale developed by Refs.^40^ and ^41^, respectively. All items were used on a 5-point Likert scale ranging from “strongly disagree” to “strongly agree”.

### Behaviral analysis

Vohs et al. has reported that people who overspend tend to purchase products impulsively^42^. Based on this research, we compared the impulse buying tendency between Sessions 1 and 2 by calculating the sum of the products purchased by the participants. We further calculated the total number of purchased products for each category to understand the difference in impulse buying tendencies based on the participants' preferences.

### Statistical analysis

All statistical analyses were performed using the SPSS version 25 (SPSS Inc., Chicago, IL, USA). Variables were examined for normality, M, s.d., and s.e.m, and then they were calculated separately for each Session or ROI region. We conducted statistical analyses of fNIRS signal values and self-report measurement responses.

Levene’s test was conducted to determine the homogeneity of variance, and then we used a two-sample t-test to analyze the differences of fNIRS signals between Sessions 1 and 2. Moreover, we tested using a one-way analysis of variance (ANOVA), with a significance threshold of $p<0.05$ to investigate the fNIRS signal differences in four ROI regions. For multiple comparisons of four ROIs, a Scheffe correction, known as the most conservative and rigorous post hoc test^43^, also showed a p-value of $<0.05$.

Furthermore, we manipulated the time pressure (high or low conditions) and price discount (availability of discounts), and then calculated the difference in self-reported scores using an independent t-test. Additionally, one-way ANOVA and one-way ANCOVA were used to calculate the score differences between impulse and non-impulse buying behaviors in the self-reported measurement and to calculate the Cronbach's alpha (α) to assess the internal consistency of self-reported measurements. α ranges from 0 to 1, with higher values indicating that self-reported measurements are more reliable.

### Informed consent statement

Informed consent was obtained from all subjects involved in the study, as described in “Methods”.

### Institutional review board statement

The study was conducted according to the guidelines of the Declaration of Helsinki, and approved by the Institutional Review Board of Korea University (KUIRB-2022-0127-01, 20 April 2022).

## Results

### Manipulation check

In this study, the reliability of self-reported measurements was validated above 0.7 ($\alpha$) by calculating impulse buying behavior ($\alpha =0.872$), time pressure ($\alpha =0.745$), and price discount ($\alpha =0.806$). These results ensure the reliability of self-reported measurements for three states. To manipulate the time pressure as an independent variable, we divided it into high and low conditions. Participants reported a significant difference between the high ($\mathrm{M}=4.48,\mathrm{ s}.\mathrm{d}.=0.95$) and low conditions ($\mathrm{M}=3.19,\mathrm{ s}.\mathrm{d}.=0.90;\mathrm{F}\left(1, 58\right)=0.06, ^{*}p<0.001$) in terms of time pressure, which suggests that our manipulation of time pressure was successful. In Session 1 with a discount of 30%, participants perceived that the discount was greater ($\mathrm{M}=5.31,\mathrm{ s}.\mathrm{d}.=0.80$) than that of Session 2 ($\mathrm{M}=4.04,\mathrm{ s}.\mathrm{d}.=1.29;\mathrm{F }\left(1, 58\right)=10.60, ^{*}\mathrm{p}<0.05)$.

### Self-reported results

One-way ANOVA was conducted by setting impulse buying behavior as a dependent variable, and online shopping sites as an independent variable (online duty-free shops vs. online shopping malls). Impulse buying behavior in online duty-free shops ($\mathrm{M}=4.52,\mathrm{ s}.\mathrm{d}.=1.35$) is higher than impulse buying behavior in online shopping malls $(\mathrm{M}=3.26,\mathrm{ s}.\mathrm{d}.=1.46;\mathrm{F}\left(1, 58\right)=12.02, ^{*}\mathrm{p}<0.05).$

One-way ANCOVA analysis was performed with gender as a covariate to check whether gender has an effect on these results. We found gender had no significant effect on impulse buying behavior (p = 0.612).

### Behavioral results

In the two-session analysis, the total response count of Session 1 ($\mathrm{M}=7.13,\mathrm{ s}.\mathrm{d}.=3.27$) was higher than that of Session 2 ($\mathrm{M}=5.33,\mathrm{ s}.\mathrm{d}.=2.34$); this result means that participants shopped more during Session 1 than in Session 2. There are statistical differences between the two sessions, resulting in a larger number of products purchased during Session 1 than in Session 2 ($t=2.372, ^{*}\mathrm{p}<0.05$).

Specifically, the sum and standard deviation of the products purchased during Session 1 were cosmetics ($\mathrm{SUM}=47,\mathrm{ s}.\mathrm{d}.=1.05$), alcohol ($\mathrm{SUM}=60,\mathrm{ s}.\mathrm{d}.=1.26$), health supplements ($\mathrm{SUM}=46,\mathrm{ s}.\mathrm{d}.=0.99$), cigarettes ($\mathrm{SUM}=19,\mathrm{ s}.\mathrm{d}.=0.60$), and perfumes ($\mathrm{SUM}=42,\mathrm{ s}.\mathrm{d}.=0.84$). Similarly, Session 2 corresponds to cosmetics ($\mathrm{SUM}=38,\mathrm{ s}.\mathrm{d}.=0.63$), alcohol ($\mathrm{SUM}=40,\mathrm{ s}.\mathrm{d}.=0.91$), health supplements ($\mathrm{SUM}=37,\mathrm{ s}.\mathrm{d}.=0.67$), cigarettes ($\mathrm{SUM}=17,\mathrm{ s}.\mathrm{d}.=0.62$), and perfumes ($\mathrm{SUM}=28,\mathrm{ s}.\mathrm{d}.=0.44$). Thus, the significant differences in the sum of products purchased between Session 1 and Session 2 were observed in categories of alcohol ($t=2.307, ^{*}\mathrm{p}<0.05$) and perfumes ($t=2.646, ^{*}\mathrm{p}<0.05$). The difference in the sum of products purchased in cosmetics ($t=1.316,\mathrm{ N}.\mathrm{S}.$), health supplements ($t=1.352,\mathrm{ N}.\mathrm{S}.$), and cigarettes ($t=0.416,\mathrm{ N}.\mathrm{S}.$) was not significant.

### Behavioral results

**Figure 3:**
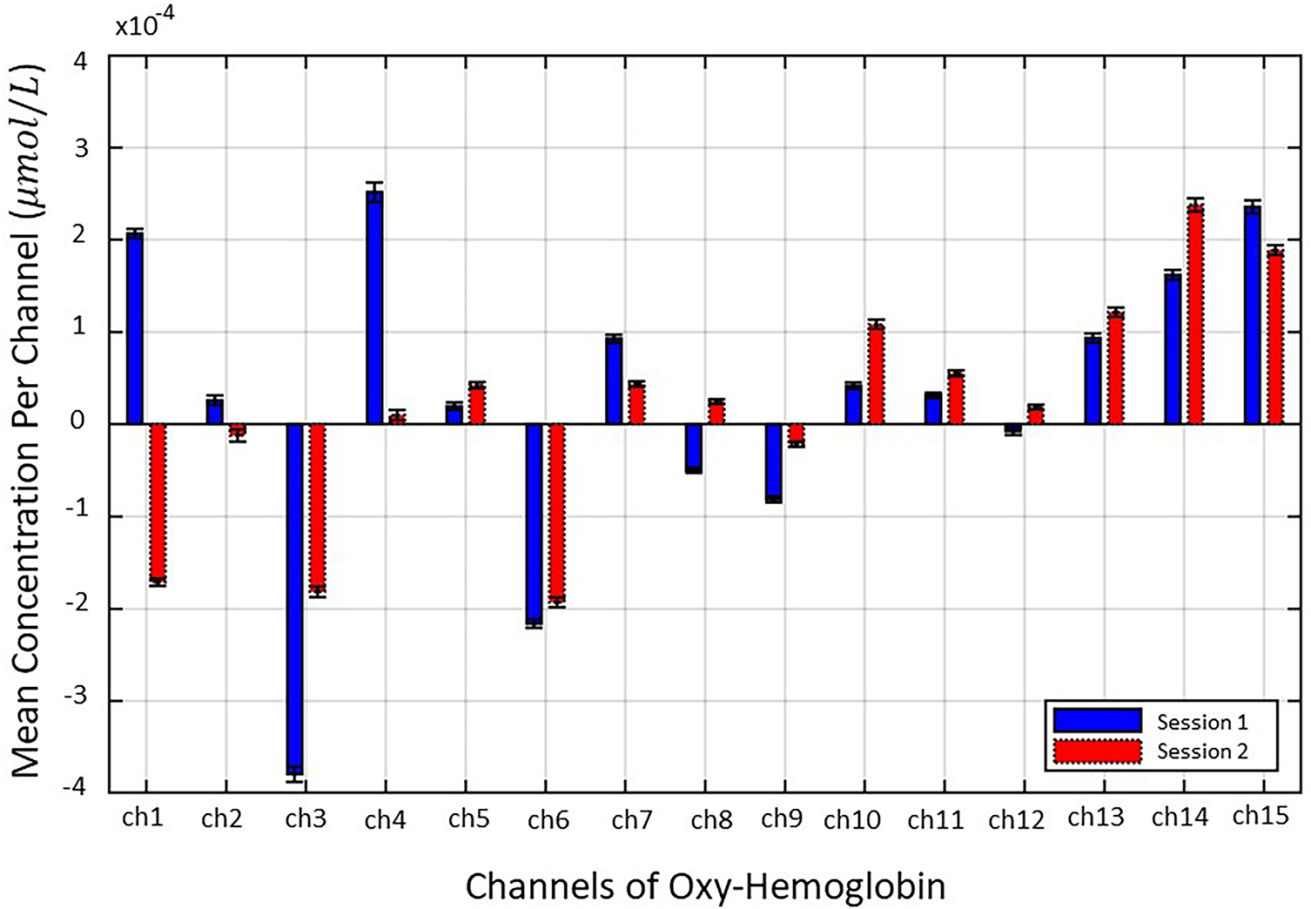
Average concentration of HbO for each channel. The blue bars are Session 1 and red bars refer to Session 2. Error bars indicate standard error of the mean (s.e.m) to estimate the variability of the mean concentration of HbO per channel. Stars indicate a statistically significant differences in mean concentration of HbO between Session 1 and Session 2 conditions $$(^{***}<0.001; ^{**}p<0.01)$$. In all channels, hemodynamic differences between the two sessions are markedly different. This suggests the possibility of judging people's impulse buying tendencies.

To quantify the brain activity patterns, we calculated the mean concentrations of ΔHbO between Session 1 (blue bars) and Session 2 (red bars) per channel as illustrated in Fig. 3. In all channels on the right (Chs. 1, 2, 3, 4, 6, and 7) except for Channel 5, the mean concentration of ΔHbO in Session 1 exhibited a higher positive or negative activation than in Session 2. Similarly, except for channel 9 and 15, the left channels from Chs. 10 to 14 exhibit higher neural activity in Session 2 than in Session 1. This phenomenon is explained by the hemispheric asymmetry theory^44^. This theory says that the higher the impulsivity, the more the frontal activity is biased to the right. Here, the frontal activity refers to the quantified mean concentration of ΔHbO. Hence, we can predict people’s impulse buying behavior through biased right frontal activities in Session 1. Moreover, we found statistically significant differences in the mean concentration of ΔHbO using a two-sample t-test, indicating that the two sessions can be distinguished. The stars show a statistically significant difference in the mean concentration of ΔHbO between both sessions (***$$p<0.001; ^{**}p<0.01$$). The error bars refer to the s.e.m to estimate the variability of the mean concentrations of ΔHbO. Eventually, these results not only show that our experimental design is well designed to elicit impulse buying behavior, but also provide evidence as a potential biomarker to distinguish between impulse buying behavior and non-impulse buying behavior with fNIRS signals.

### Difference of ∆HbOs between impulse buying behavior and non-impulse buying behavior

**Figure 4:**
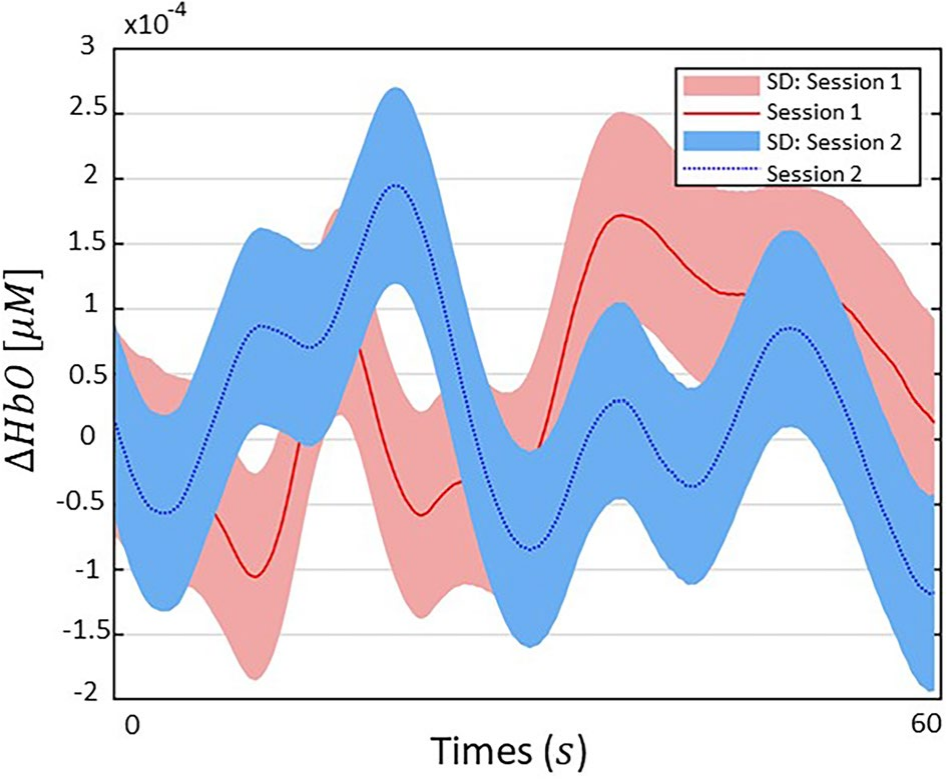
Hemodynamic responses for the averaged change concentration of ∆HbO between Session 1 and Session 2. The ∆HbO represents the average interval from the start to the end of the task (0–60 s) acquired from 30 participants. The solid red line and dotted blue line are ∆HbOs of Session 1 and Session 2, respectively. The corresponding shaded colors are the standard deviations (s.d.) from the mean signals. The hemodynamic signals between the two sessions are markedly different ($$t=3.297; ^{***}p<0.001$$), indicating the role of biomarkers for those who show impulse buying patterns.

Figure 4 illustrates the ∆HbOs depending on the average interval (0–60 s) from the start to the end of the tasks. In this experiment, the ∆HbOs are average hemodynamic signals across all participants to compare Session 1 (red lines) shopped in an online duty-free shop environment with Session 2 (blue lines) shopped in an online shopping mall environment. Their s.d. is expressed in red shading in Session 1 and blue shading in Session 2, respectively. The signal difference between the two sessions is significant ($$t=3.297, ^{***}p<0.001$$) statistically, indicating that the ∆HbOs gap between the two sessions is enormous. Thus, this finding can be used to forecast impulse buying trends.

### Detect of impulse buying patterns using SVM, the popular artificial intelligence algorithm

**Figure. 5:**
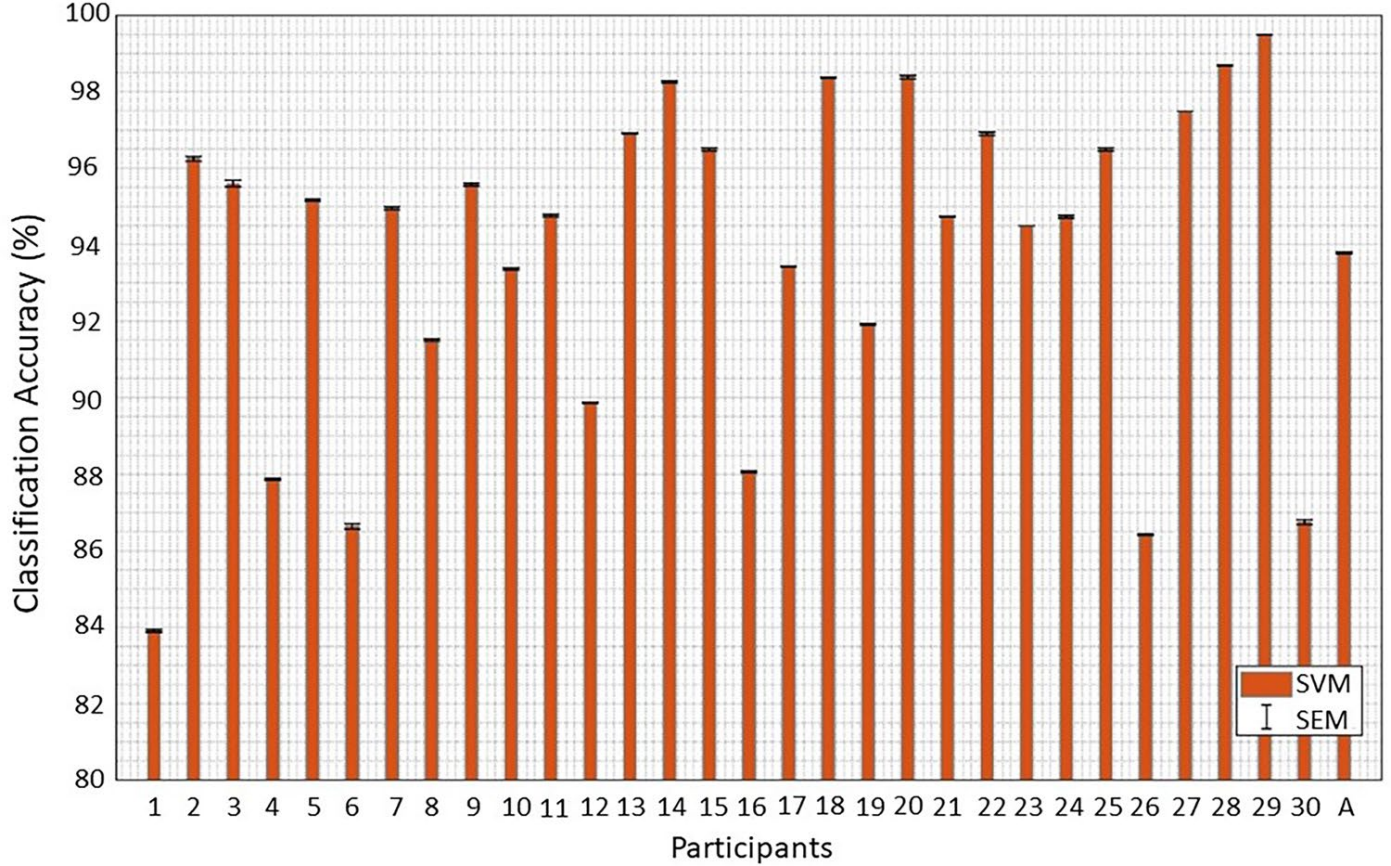
Classification accuracies of individual subject and grand averaged classification accuracy between Session 1 (impulse buying) and Session 2 (non-impulse buying) using SVM. All participants show the accuracies in the range of 83.91–99.49%. These results suggest that fNIRS data can be used as a biomarker to differentiate between impulse and non-impulse buying behaviors in terms of the BCI technology.

Figure 5 illustrates the objective evidence for detecting impulse buying behaviors. Specifically, the classification accuracies of individual subject and grand averaged classification accuracy are represented well between Session 1 (impulse buying) and Session 2 (non-impulse buying) using SVM. ‘A’ in the x-axis indicates the average classification accuracy across the 30 participants with an average accuracy of 93.78% and a s.e.m of 0.03 for detecting impulse buying patterns. Furthermore, the classification accuracies are also provided for each participant. The accuracies of all the participants are ranged from 83.91 to 99.49%. As a result of these findings, fNIRS data can be used as a biomarker to distinguish between impulse and non-impulse buying behaviors in terms of the BCI technology.

### Oxygenated hemoglobin activation based on ROIs within PFC

**Figure. 6:**
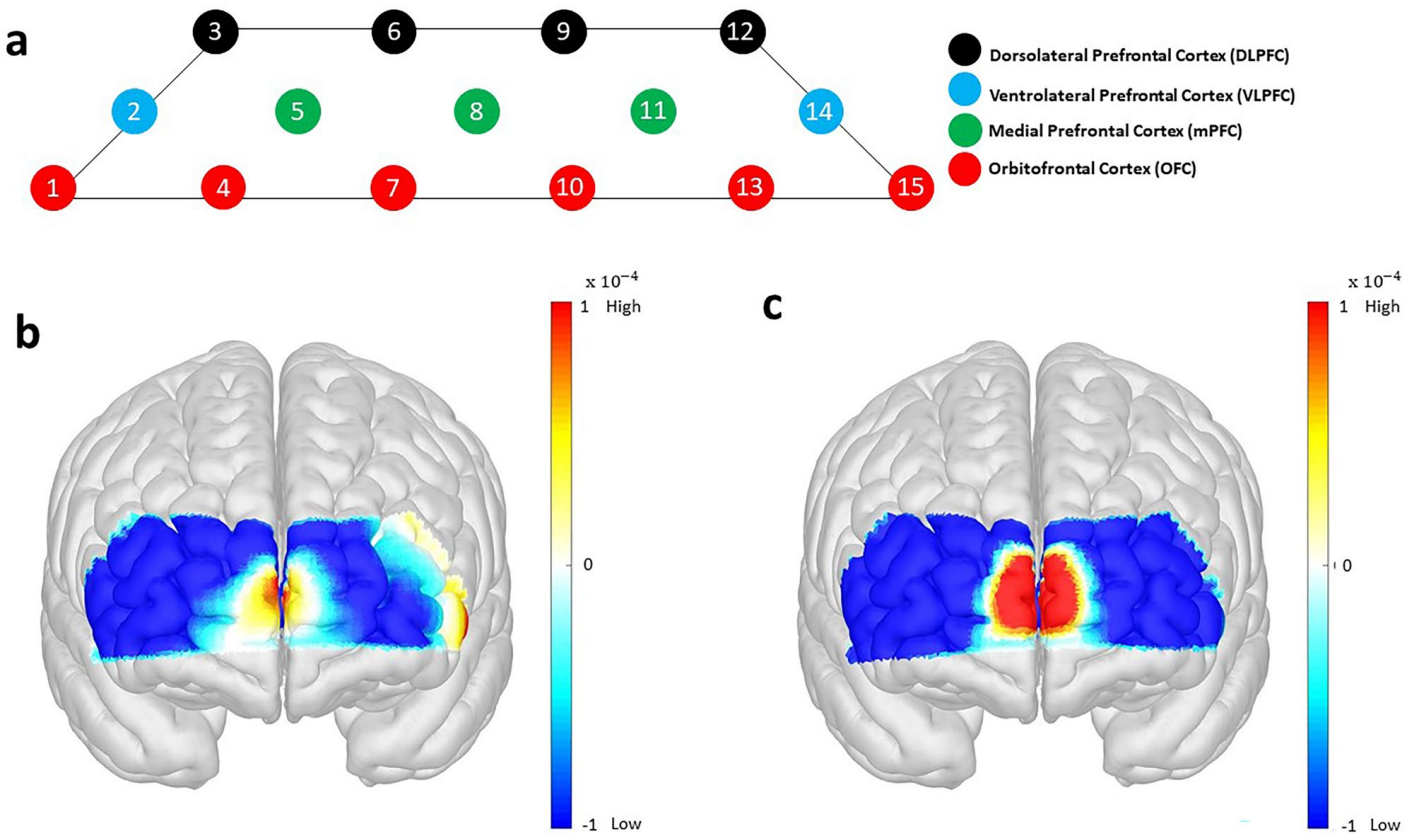
Topographical maps of averaged ∆HbO activation at the PFC for 30 people: (**a**) shows a configuration of ROIs based on the Montreal Neurological Institute coordinates and their associated channels with the arrangement of 15 channels covering the forehead. (**b**,**c**) are averaged ∆HbO corresponding to Session 1 and Session 2, respectively. The color bars refer to the quantified brain activation within the range − 1 (low) to 1 (high). Session 1 shows the neural activity in mPFC, which is insignificant but active in the left VLPFC (Chs. 12, 14, 15). However, Session 2 illustrates a high activation in a wide range over the DLPFC, mPFC, and OFC except for the VLPFC region. The activation of mPFC and left VLPFC stems from the reward-related mechanisms, but the other areas are known to be responsible for inhibiting impulsiveness. This phenomenon is also statistically expressed, and a distinct ∆HbO difference has also been found between mPFC and the other areas ($$^{***}p<0.001$$). In other words, we believe that reasonable consumption is possible because it inhibits people's impulse buying behavior in the online shopping process, unlike online duty-free shopping.

Figure 6 illustrates the topographical maps of the averaged ∆HbO activation at the PFC across all participants. Figure 6a presents the configuration of ROIs in accordance with the Montreal Neurological Institute, representing the template adopted by the international consortium for brain mapping. The ROIs consist of the upper DLPFC (black circles), VLPFC on both sides (blue circles), the middle mPFC (green circles), and the bottom OFC (red circles). Furthermore, Fig. 6b,c present the topographical maps for the hemodynamic activations of Session 1 and Session 2, respectively. The color bars represent the quantified brain activation from -1 (low activations) to 1 (high activations).

In Fig. 6b, Session 1 elicits significant neural activity in multiple areas. Especially, major clusters of activities were found within the mPFC. The mPFC is closely related to the reward mechanisms; the higher the expectation for reward, the more prominent is the activation of the mPFC. Statistically, the activated contrast between mPFC and the other areas is presented using ANOVA ($$F=1106.396; ^{***}p<0.001$$), which indicates that the difference in neural activation between the mPFC and the remaining areas is large. We also observed that the left VLPFC was weak yet activated and this area is related to the reward systems. VLPFC is mainly involved in impulse suppression, however, the VLPFC on the right and left sides have different functions in detail. Especially, the higher the reward expectancy, the higher is the activation of the left VLPFC. In contrast, there is a minor change in other areas dealing with impulse suppression. Thus, it is not easy to regulate impulse buying behavior in an online duty-free shop environment (Session 1).

Compared with Figs. 6b,c indicates that a high neural activity was observed in all areas except for VLPFC located on both sides of the forehead. It is interpreted that the reward sensitivity is high because of the activated mPFC region, but Session 2 can regulate impulse buying behavior relatively better than Session 1. This is because it exhibits high activity in the DLPFC and OFC areas, which indicates that the impulse suppression mechanism is functioning properly. There is a significant activated difference in fNIRS signals obtained between mPFC and other areas ($$F=443.237; ^{***}p<0.001$$), which can be distinguished between mPFC responsible for the reward mechanisms and the remaining areas responsible for impulse suppression activity. Therefore, it suggests that reasonable consumption is possible because it inhibits the subject's impulse buying behavior in the online shopping process, unlike online duty-free shopping.

## Discussion

Marketing literature focuses on developing a model to explore and predict consumers’ purchasing decisions impulsively^45^. However, the validity of the model adapted through existing marketing tools is controversial. Existing studies indicate that traditional methodologies such as self-report inventory, surveys, or interviews are not enough at predicting actual impulse buying behavior^12^.

However, we can solve the aforementioned problems by the BCI technology using fNIRS. Figures 3, 4, 5 and 6 indicate that there is a significant difference in the mean concentration of ∆HbO between the manipulations that evoked impulse buying and non-impulse buying behaviors. These findings are consistent with previous research, which found that fNIRS can not only reduce the biases caused by conscious thoughts measured by self-reports but also ensure the ecological validity of neurophysiological models of people’s behavior^12^. The novel technique using fNIRS enables researchers to capture shoppers’ unconsciousness^46^, which is difficult to be reflected in a verbalized manner such as through self-reports, surveys, or interviews. Thus, we proposed a neuromarketing approach that combines brain neurophysiological methodology and marketing methodology to solve the problems of traditional marketing methodologies.

fNIRS signals have the potential to be biomarkers that distinguish impulse buying behavior from non-impulsive buying behavior. The need for biomarkers is gradually emerging because they can capture the human impulse buying tendencies instantly by supplementing traditional measurement tools. The emergence of fNIRS-based biomarkers has increased the potential of impulse purchase detection, which can improve prediction on the human purchase decision-making. Recently, some studies have reported the potential of biomarkers by neuroimaging methods to explain human intuitive tendencies that are difficult to express with traditional methodologies alone^47^. A recent study has reported that the buy/no-buy decision can be predicted accurately using neural activity patterns obtained from fMRI^48^. Several studies propose neural evidence for the involvement of socio-cognitive processes between advertising effects and advertising memory based on fMRI and fNIRS^49^. These results are consistent with our findings. We also applied the innovative neuroimaging method of fNIRS to explore these unconscious neural perception processes for Session 1 and Session 2 as illustrated in Figures 3 and 4. Furthermore, Fig. 5 represents classification accuracy (93.78%) when detecting impulse buying behavior using fNIRS-SVM, which was attempted for the first time to the best of our knowledge. Current research has shown that activation of PFCs can serve as objective neural biomarkers, focusing on the analysis of impulse or non-impulsive purchase tasks through fNIRS.

We have adopted marketing concepts to more dramatically differentiate impulse and non-impulsive purchases. Specifically, humans prefer to receive fast but small rewards rather than slow but large rewards^50^. This tendency is often described by models called temporal discounting or delay discounting. Temporal discounting refers to devaluing temporally distant rewards. Thus, this concept is often used as a measure of impulsiveness or expectations for rewards. According to a previous fMRI study^51^, temporal discounting has been shown to be closely related to the mPFC region, and the brain information regarding reward and delay might be combined in this region. As presented in Fig. 6, our results are identical to the previous study results. In this study, we instructed the participants to purchase the product within a short period of time. In the process, the participants exhibited strong impulse buying tendencies that only represent mPFC activity, unlike when they shopped online freely regardless of time.

Conversely, when participants shop freely online regardless of time, the OFC and DLPFC activities were also observed along with the mPFC. They govern the suppression of impulsiveness. A study reported a case indicating that patients with borderline personality disorders have decreased activation of the OFC when compared to healthy people^52^. Another study provided evidence that the DLPFC could play a specific role in inhibitory control of decision-making, especially impulsive decision-making related to compensatory value and time perception^53^. Therefore, we believe that this neuroscientific evidence will help to distinguish impulse buying patterns empirically.

One of the factors that separates the impulse buying pattern may be visual aesthetic stimulation. Several studies have reported that travelers' impulse buying patterns are affected by the interior and atmosphere of offline duty-free shops^54^. The same effect can be obtained by designing a user interface (UI) in online website environments^55^. Specifically, some studies have demonstrated that an attractive online website design encourages impulse buying behavior in shoppers^56^. Customers who are more interested in an appealing website design and user-friendly online services regard shopping as a recreational activity. They are more interested in the shopping process than in completing the shopping. In this process, customers’ impulse buying behaviors are increased exponentially. Our result suggests the possibility of encouraging impulse buying in online duty-free shopping platforms. Our study found that the total number of items purchased by participants on purchase screens for online duty-free shops was overwhelmingly high. This is attributed to visual aesthetics on the website^42^. We additionally conducted a google survey to investigate whether the website designs used in our study are visually aesthetic^57^. We showed 73 people both the duty-free and general shopping mall websites used in this experiment, and then we asked them to select the most visually appealing screen. As a result of the online survey, the respondents answered at the following rates: “Only duty-free website is aesthetic” (46.6%), “Only online shopping mall website is aesthetic” (30.1%), “both aesthetic” (4.1%), “Neither aesthetic” (19.2%). In the end, most respondents perceived aesthetics in the design of the online duty-free website used in this experiment, and therefore encouraged impulse buying.

To empirically analyze these phenomena, there have recently emerged neuroimaging techniques including fNIRS to monitor the brain interactions by the visual stimulations. The PFC is the most important area in the human brain and supervises the formation and manipulation of mental images and coordinates in spatial and sensory areas^58^. Many studies have shown that the designs of websites have a great influence on brain activity as measured by fNIRS^59^. A study showed a user-friendly website screen and a user-unfriendly website screen and then used a fNIRS to detect brain PFC activity^60^. Another study was discovered that visual commercialization enhanced consumers' impulse buying behavior, resulting in a change in brain activation^61^. Therefore, website designs influence impulse buying behavior by providing powerful visual stimulation, which has a significant influence on PFC activation, and brain activation for specific impulse buying behavior can be observed using an fNIRS.

## Conclusion

Our study shows whether a multidisciplinary approach with BCI technology through brain signal processing, as well as self-reported measurement, is necessary to prove the effectiveness of human impulse buying behavior. It provides a starting point for exploring future research efforts in the field of BCI technology. In this case, it was revealed that the joy of the travel, time pressure, and product discount price affect impulse buying behavior. In conclusion, this study demonstrated that brain activation differs between impulse buying behavior and impulse suppression situations. We also showed that activation of PFCs can serve as objective neuronal biomarkers focused on impulsive or non-impulsive analysis. Moreover, we used SVMs for 30 people to achieve average accuracy for impulse buying behavior (93.78%). Concisely, this study empirically shows that the BCI technique can enable us to gain more insight and understand people’s impulsive behavior by supplementing traditional measurement tools such as human self-reported.

## Data Availability

Data of our study are available upon request. Requests for materials should be addressed to S.B.
